# Transcriptional regulation of the paper mulberry under cold stress as revealed by a comprehensive analysis of transcription factors

**DOI:** 10.1186/s12870-015-0489-2

**Published:** 2015-04-19

**Authors:** Xianjun Peng, Qingqing Wu, Linhong Teng, Feng Tang, Zhi Pi, Shihua Shen

**Affiliations:** Key Laboratory of Plant Resources, Institute of Botany, the Chinese Academy of Sciences, Beijing, 100093 People’s Republic of China; University of the Chinese Academy of Sciences, Beijing, People’s Republic of China

**Keywords:** Cold stress, Plant development, Signaling crosstalk, Transcription factor

## Abstract

**Background:**

Several studies have focused on cold tolerance in multiple regulated levels. However, a genome-scale molecular analysis of the regulated network under the control of transcription factors (TFs) is still lacking, especially for trees. To comprehensively identify the TFs that regulate cold stress response in the paper mulberry and understand their regulatory interactions, transcriptomic data was used to assess changes in gene expression induced by exposure to cold.

**Results:**

Results indicated that 794 TFs, belonging to 47 families and comprising more than 59% of the total TFs of this plant, were involved in the cold stress response. They were clustered into three groups, namely early, intermediate and late responsive groups which contained 95, 550 and 149 TFs, respectively. Among of these differentially expressed TFs, one bHLH, two ERFs and three CAMTAs were considered to be the key TFs functioning in the primary signal transduction. After that, at the intermediate stage of cold stress, there were mainly two biological processes that were regulated by TFs, namely cold stress resistance (including 5 bHLH, 14 ERFs, one HSF, 4 MYBs, 3 NACs, 11 WRKYs and so on) and growth and development of lateral organ or apical meristem (including ARR-B, B3, 5 bHLHs, 2 C2H2, 4 CO-like, 2 ERF, 3 HD-ZIP, 3 YABBYs, G2-like, GATA, GRAS and TCP). In late responsive group, 3 ARR-B, C3H, 6 CO-like, 2 G2-like, 2 HSFs, 2 NACs and TCP. Most of them presented the up-regulated expression at 12 or 24 hours after cold stress implied their important roles for the new growth homeostasis under cold stress.

**Conclusions:**

Our study identified the key TFs that function in the regulatory cascades mediating the activation of downstream genes during cold tress tolerance in the paper mulberry. Based on the analysis, we found that the AP2/ERF, bHLH, MYB, NAC and WRKY families might play the central and significant roles during cold stress response in the paper mulberry just as in other species. Meanwhile, many other TF families previously reported as involving in regulation of growth and development, including ARF, DBB, G2-like, GRF, GRAS, LBD, WOX and YAABY exhibited their important potential function in growth regulation under cold stress.

**Electronic supplementary material:**

The online version of this article (doi:10.1186/s12870-015-0489-2) contains supplementary material, which is available to authorized users.

## Background

The paper mulberry (*Broussonetia papyrifera*) belongs to the family of Moraceae and is naturally distributed in Eastern Asia and pacific countries. The paper mulberry has the shallow roots morphology with advanced lateral roots but without an obvious taproot. The paper mulberry is one of the multifunctional tree species in agroforestry systems [1], as well as being one of the traditional forages [2] and Chinese medicines in many countries of Asia [3]. It is the ideal tree species for ecological and gardening purposes [4]. Due to its fast growth and adaptability, the paper mulberry is commonly used for the ecological afforestation and landscape in both sides of highway, mined areas and on barren land [5]. However, the molecular mechanism of strong adaptability and tolerance to biotic or abiotic stress of the paper mulberry has not been studied, which limits the exploitation of the paper mulberry.

In recent years, many reports have provided new and exciting information that has allowed us to better understand the genes involved in cold adaptation and freezing response [6]. One of the major advances in the past decade of cold tolerance research is the discovery of the cold stress related TFs. Many TFs, including DREB [7], MYB [8], NAC [9] and WRKY [10], have been found to be involved in the cold response of plants. Recently, more and more researches concentrate on TF families involved in abiotic stress including cold stress response based on genome wide analyses, such as WRKY [11], NAC [12,13], MYB [14] and AP2/ERF [15]. However, there are few reports on the role in cold responses of other TF families, such as ARF, E2F, GRF and GRAS, though their functions in development and growth have been characterized. Moreover, most studies about cold stress in plants concentrate on a few species, such as Arabidopsis [16,17] and some crops [18]. The universality of the mechanism under TF control is not explicit, especially for tree species. Because of low domestication, open-pollinated native populations and high levels of genetic variation, trees are ideal organisms to unveil the molecular basis of population adaptive divergence in nature and have gained much attention in recent years as non-classical model plants for environment adaptation, evolutionary and genomic studies [19]. A substantial number of ESTs (expressed sequence tags) encoding putative transcription factors, including CBFs (C-repeat binding factor), are observed in *Eucalyptus* under low temperature [20]. In subgroup III of the *PtWRKY* genes in *Populus*, eight were induced expression under cold stress [21]. A comprehensive analysis of the NAC gene family in *Populus* revealed the functional divergence among members in NAC family [22]. Over-expression of *JcERF* or *JcDREB*, isolated from *Jatropha curcas*, a woody oil plant, in transgenic Arabidopsis exhibited enhanced salt and freezing stress responses [23,24]. *BpDREB2*, cloned from the paper mulberry, could enhanced the freezing tolerance of Arabidopsis significantly without causing growth retardation [25]. Genetic diversity revealed by SRAP (Sequence-related amplified polymorphism) marker and cluster analysis show that there is a relationship between the genetic variation and geographical distribution [26]. These results provide reference for making genetic map and guiding the breeding of the paper mulberry. In this study, we discern the potential transcriptional regulatory network regulated by the TFs in the seedlings of the paper mulberry from transcriptomic data under cold stress.

## Results and discussion

### Identification and classification of TFs in the paper mulberry under cold stress

TFs play the significant roles in plant development and stress tolerance. To identify the TFs involved in the cold stress response, we surveyed the biological functions of putative TFs that were differentially expressed in the paper mulberry under cold treatment. After retrieving annotation results for every unigene, there were total of 1,337 TFs in the paper mulberry and classified into 55 families based on their DNA-binding domains and other conserved motifs [27]. The expression level of all TFs was normalized by using their RPKM values. Among of these, total of 1,180 TFs, belonging to 52 families, were identified as being expressed in paper mulberry during cold stress (Additional files 1 and 2A). Of these, 441 TFs had the complete ORF (opening read frame) representing 37.02% of the total (Additional file 3).

**Figure 1:**
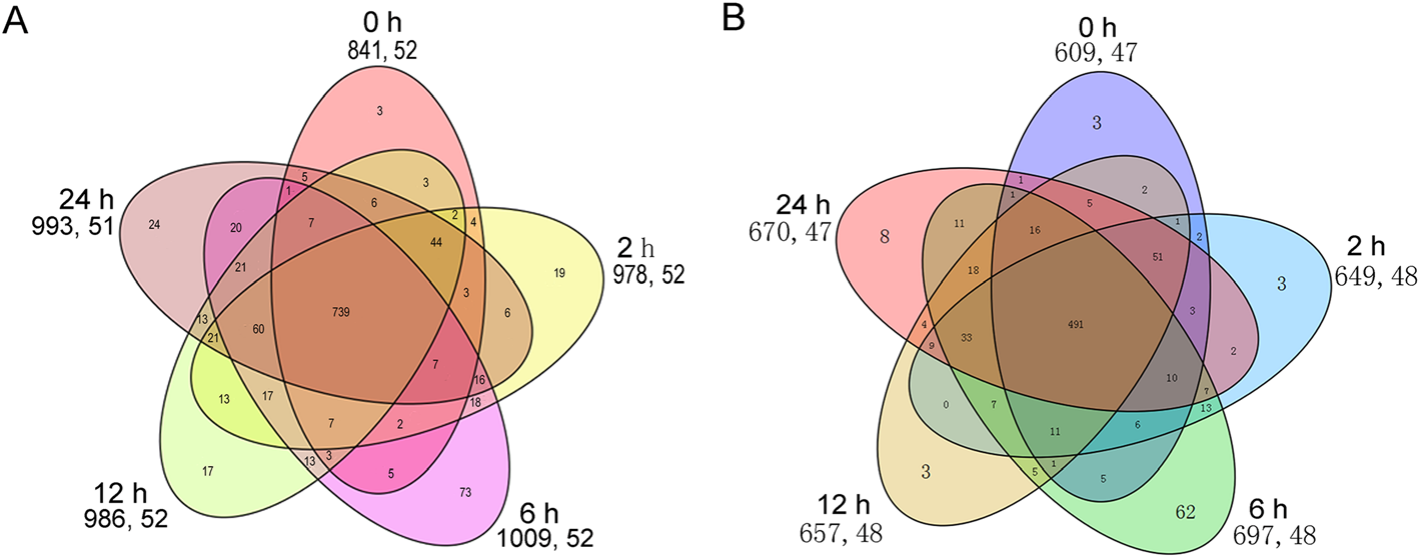
No. of expressed and differentially expressed TFs in each sample. **A** Venn diagram of expressed TFs distributed in five samples **B** Venn diagram of differentially expressed TFs in every sample. The time was represented the sample that were treated under cold stress. The former and latter number presented below the treat time, such as (841, 52), was the number of TFs and families presented in this sample, respectively.

Under non-stress conditions, 841 TFs belonging to 52 families were expressed in the leaves. This number reached 978 after 2 hours of cold stress and 1,009 after 6 hours. When 12 hours passed under low temperature, the number of expressed TFs was reduced to 986. After 24 hours of exposure to cold, the number of expressed TFs was 993 (Figure 1A). A total of 739 TFs were expressed in all five samples, representing 62.04% of the total TFs. A relatively small number of TFs were uniquely expressed in each sample: 3, 19, 73, 17 and 24, respectively.

### Differentially expressed TFs the paper mulberry under cold stress

Temperature is an important environmental factor that affects plant growth and development. Temperature stresses include cold, freezing and heat. Expression data from different plants species have indicated that the members of the TFs participate in plant responses to cold stresses. After four hours of cold treatment, a total of 70 up-regulated and 18 down-regulated TFs were identified in the well-developed leaf of *Vitis amurensis* while 68 up-regulated and 43 down-regulated TFs were identified in *Vitis vinifera* [28]. The transcripts of 35 TFs from 6 TF families were responsive to cold stress in *Populus simonii* [29]. When *Ammopiptanthus mongolicus,* a desert shrub, was cultured at 4°C for 14 days, a total of 720 TFs were identified as DEGs, 209 of which showed significant up-regulation and 511 down-regulation [30]. In our study, a total of 794 TFs were responsive to cold stress, representing 59.38% of the total number of TFs expressed in the paper mulberry (Figure 1B and Additional file 4). 491 TFs were expressed in all five samples, representing 61.84% of the total differentially expressed TFs. The number of specifically expressed TFs was 3, 3, 62, 3 and 8, respectively. These results show that the number of TFs involved in cold response varies considerably among different tissues of different species under different conditions. This implies that the individual plant species have distinct cold response.

**Figure 2:**
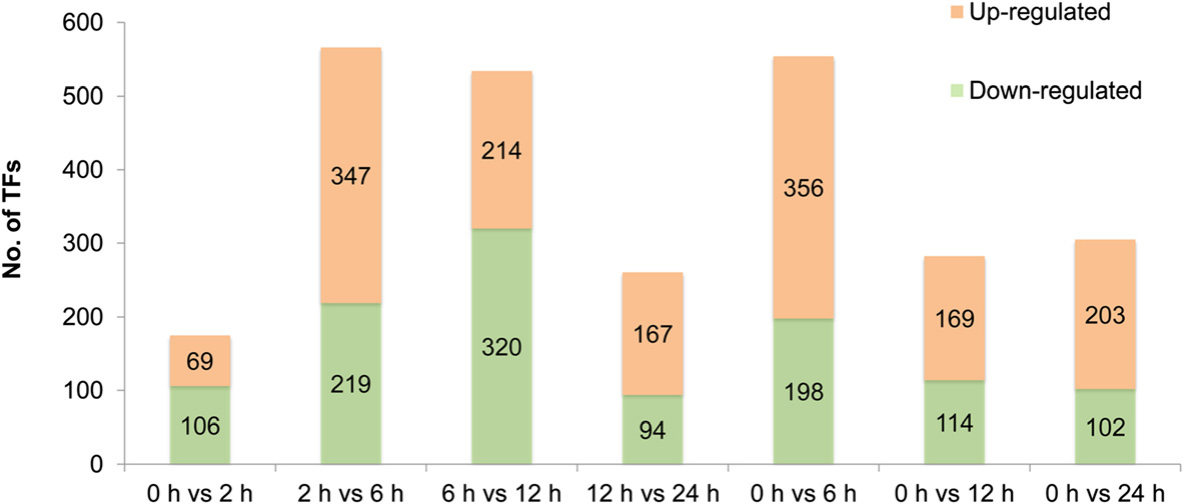
Cold stress responsive TFs between every two samples. Taken pairwise comparison of 0 h vs 2 h as an example, up-regulated was referred to the expression was higher than that after 2 hours of cold stress treatment while down-regulated referred to the expression was lower than that after 2 hours of cold stress treatment.

After 2 hours of cold stress treatment, 175 TFs showed differential expression characteristics, of which 69 TFs were up-regulated and 106 were down-regulated (Figure 2). The number of repressed TFs was significantly more than the number of induced expression TFs. Compared with the untreated leaves, a total of 564 TFs were differentially expressed after 6 hours of cold stress treatment, of which 356 were up-regulated and 198 down-regulated TFs. Meanwhile, there were 566 differentially expressed TFs between 6 hours and 2 hours after cold stress treatment, with 347 up-regulated TFs and 219 down-regulated. After 12 hours of cold stress, the number of differentially expressed TFs (273) decreased dramatically compared with the untreated leaves, with 169 up-regulated and 114 down-regulated TFs. However, when comparing gene expression at 12 hours of cold exposure to 6 hours, there is a significant increase in the number of down-regulated TFs (320), which is more than the number of up-regulated TFs (214). This suggests that most of the TFs up-regulated at 6 hours were reduced upon another 6 hours of exposure to cold, and thus are only transiently activated in response to this stress. After 24 hours of cold stress treatment, there were 261 differential expression TFs, including 167 up-regulated and 94 down-regulated TFs when compared with 12 hours of treatment.

To validate the transcriptomic data, we selected 10 TFs for confirmation by qPCR (Additional file 5). The qPCR results suggested that the expression profile of the TFs were consistent with the transcriptomic data, though there was no biological replicate in the transcriptomic experiment. This was mainly benefit from using the clonal seedlings in this study.

### Family distribution of the differentially expressed TFs

**Figure 3:**
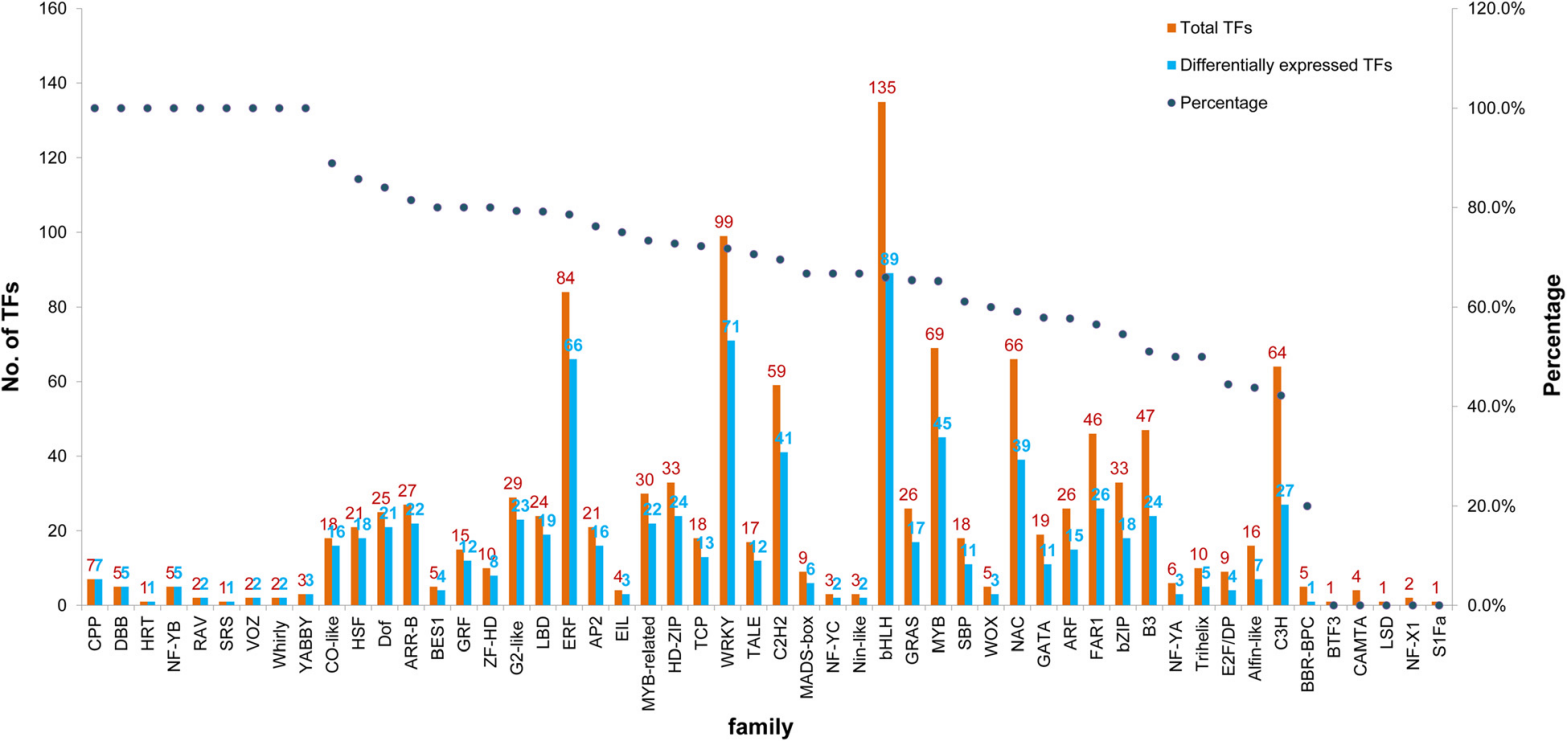
Total and cold stress responsive TFs distributed in every family. “Total TFs” referred to the total number of TFs in each family expressed in the leaf of the paper mulberry. “Differentially expressed TFs” referred to the TFs with dramatic change in the leaf of the paper mulberry under cold stress. “Percentage” referred to the percentage of the “Differentially expressed TFs” number accounting for the “Total TFs” number of each family.

A total of 794 differentially expressed TFs could be classified in to 487 families (Figure 3 and Additional file 2B). The bHLH family contained the most differentially expressed TFs (89), followed by WRKY (71), ERF (66), MYB (45), C2H2 (41) and NAC (39). Regarding the CPP (7), DBB (5), HRT (1), NF-YB (5), RAV (2), SRS (1), VOZ (2), Whirly (2), YABBY (3) families, all known members were responsive to cold stress. Meanwhile, all the members of BTF3, CAMTA, LSD, NF-X1 and S1Fa family did not change in expression during cold stress treatment. However, according to the results of qPCR (Additional file 5), three members of the CAMTA family showed a significant expression difference in the early stage (15 min or 30 min) of cold stress treatment though this was not detected in the transcriptomics data.

**Figure 4:**
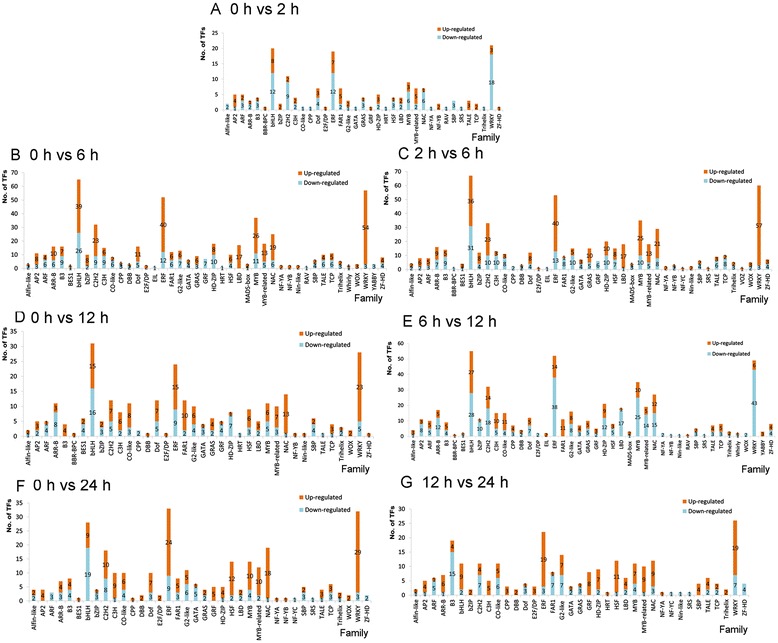
Cold stress responsive TFs distributed in each family of every pairwise comparison. **A**-**G** Family distribution in each pairwise comparison. Numbers on the column represent the amount of up-and –down regulated TFs distributed in the family.

There were 175 TFs belonging to 37 families (Figure 4A) responded to cold stress after 2 hours of treatment; most of these TFs showed a decrease in expression. For example, there were 12 down-regulated ERF family TFs compared with 7 up-regulated; 6 down-regulated NAC family members, and only one up-regulated; and 18 down-regulated and 3 up-regulated WRKY family TFs. Total 43 families are involved in the cold stress response at 6 hours of treatment (Figure 4B). Whether it is compared with the untreated sample or with the 2 hours treated sample, the up-regulated TFs in most families increased significantly, while there was little to no change in the number of down-regulated TFs. For example, the number of up-regulated TFs in the WRKY family increased to 57 and the number down-regulated reduced to 3 (Figure 4C). After 12 hours of cold stress, there were 44 TF families that exhibited differential expression. For most families, when compared with the untreated leaf sample, the number of up-regulated TFs was clearly more than down-regulated TFs (Figure 4D). However, the numbers of down-regulated TFs were significantly more than the up-regulated in the bHLH, WRKY, ERF, MYB, C2H2, NAC and LBD families when compared with 6 hours cold stress treated leaf (Figure 4E). The Whirly (1 up-regulated and 1 down-regulated), RAV (1 up-regulated), and YABBY (3 up-regulated) TF families emerged as having members with differential expression at this time, while the NF-YC and VOZ families were no longer expressed.

Compared with the untreated leaf, a total of 305 TFs belonging to 38 families were differentially expressed in the leaf treated under cold stress for 24 hours. The numbers of up-regulated TFs was significantly more than that of down-regulated TFs in most families, except the bHLH family with 9 up-regulated and 19 down-regulated TFs (Figure 4F). When compared with the leaf treated with cold stress after 12 hours, only 261 TFs belonging to 37 families showed the differential expression traits at 24 hours of cold stress. The B3 family changed greatly with 15 TFs down-regulated and 4 up-regulated. Regarding the ERF, GRF, HSF, NAC and WRKY families, the number of up-regulated TFs was obviously higher than that of down-regulated TFs. The TF family HRT (1 up-regulated) and NF-YC (1 down-regulated) emerged as having differentially regulated members after 24 hours of cold exposure, while the BBR-BPC, BES1, EIL, NF-YB, MADS-box, RAV, Whirly, WOX and YABBY families were no longer expressed (Figure 4G).

According above analysis, from 0 to 24 hours of cold stress treatment (Figure 4A-G), the expression pattern of TFs in the AP2/ERF, bHLH, MYB, NAC, and especially WRKY families experienced a dramatic change. Genes encoding 82 AP2/ERF, 89 bHLH, 41 C2H2, 45 MYB, 39 NAC and 71WRKY family members were responsive to cold stress in the paper mulberry, which comprise 46.22% of the total cold-regulated TFs in this species (Figure 3), and the number of expressed TFs from these families obviously increased after the cold treatment while the number of the remaining families was almost same within each treatment sample (Additional file 2A). This result was similar with previous studies. For instance, in the mature pollen of Arabidopsis, a total 93 TFs from eight families, including AP2/ERF, bZIP, bHLH, HD-ZF MYB, MADS, NAC, and WRKY, have been reported to be involved in the cold stress response process [31]. In apical shoots of cassava subjected to cold, a total of 32 genes were identified as early cold-responsive TFs; AP2/ERF, MYB, and GRAS were the three major TF families involved in this process, with 6, 5, and 5 TFs represented, respectively [32]. The WRKY [11], NAC [12,13], MYB [14] and AP2/ERF [15] TF families have been characterized as being involved in abiotic stress, including cold stress response, based on genome-wide analyses and many intensive researches have revealed their significant roles in the cold stress response.

Cold-inducible DREB1/CBFs, one of the subfamily in AP2/ERF superfamily, have been identified from numerous plant species, such as Arabidopsis [33], sheep grass [34], barley [35], *Prunus mume* [36], rice [18] and maize [37]. The DREB1/CBF cold response pathway play a central role in cold acclimation. DREB1/CBF proteins bind to DRE/CRT elements in enhancers and activate transcription of target cold-inducible genes, including other transcription factors, and thus bring about transcriptomic and metabolomics changes, which eventually cause cold stress responses [15,38]. The DREB1/CBF function in cold tolerance by ABA-dependent and ABA independent pathway. Meanwhile, under cold stress, an acute rise in cellular ABA levels induces the expression of downstream genes which contain ABA-responsive *cis* elements in their promoters, represented by various types of TFs, such as bZIP, MYB, and MYC. MYB proteins are a superfamily of TFs that have known regulatory roles in developmental processes and defense responses in plants [39]. The *HOS10* gene encodes an R2R3-type MYB and works as an important coordinating factor for responses to cold stress [17]. Expression of *OsMYB2* was up-regulated by cold and the *OsMYB2* overexpressing plants were more tolerant against cold stress than wild type plants [8]. Cold stimulates the activation of NTL6, a NAC family TF, which induces a subset of *PR* genes and the transgenic exhibited enhanced disease resistance [40]. This indicates that the mechanism by which these paralogous TFs mediate stress tolerance is complex in plants, though these TFs can bind to the same core recognition sequence [12,41].

Transgenic Arabidopsis plants overexpressing *GmWRKY21* shows increased tolerance to cold stress when compared with wild-type plants [42]. Recent research suggest that the three structurally related WRKY proteins, AtWRKY18, AtWRKY40 and AtWRKY60, participate in at least three phytohormone-mediated signaling pathways, including SA (Salicylic acid), JA (Jasmonic acid) and ABA (Abscisic acid) [43], Two closely related WRKY transcription factors (At*WRKY25* and *AtWRKY33*) respond to both biotic and abiotic stresses, e.g. *P. syringae*, NaCl, cold and heat [11].

Cross-talk and mutual regulations exist among these TFs. CBF2/DREB1C negatively regulates *CBF1*/*DREB1B* and *CBF3*/*DREB1A*, ensuring that their expression is tightly controlled, which, in turn, guarantees the proper induction of downstream genes and the accurate development of Arabidopsis tolerance to freezing and related stresses [44]. The cold regulation of *CBF3* involves upstream bHLH [45] and MYB [46] factors. Cold stress induces simulation of ICE1 at K393, which is critical for ICE1-mediated activation of transcription of *CBF*s and repression of *MYB15*. CBFs regulate the expression of *COR* genes that confer freezing tolerance. The expression of *CBF*s is negatively regulated by MYB15 and ZAT12 [47]. The MYB15 protein interacts with ICE1 and binds to MYB recognition sequences in the promoters of *CBF* genes, which suggest MYB15 is part of a complex network controlling the expression of *CBFs* in response to cold stress [46]. CAMTA3, one member of the calmodulin binding transcription activator (CAMTA) family, binds to the CM2 motif and works as a positive regulator of *CBF2* [48]. AtWRKY34 negatively mediates cold sensitivity of mature Arabidopsis pollen through regulating the expression of genes encoding CBF family transcriptional activators [10]. Therefore, we inferred that the AP2/ERF, bHLH, C2H2, MYB, NAC and WRKY family members interacted with ABA, JA and other of phytohormone might play the central and significant roles during cold stress response in the paper mulberry.

In addition, there were still many TFs distributed in other small TF families, such as ARF, DBB, G2-like, LBD, WOX the G2-like, GRF, LBD, WOX and YABBY (Figure 3 and Additional file 2B), even though they have been characterized primarily for their participation in the regulation of plant photo-morphogenesis, development and growth. Most members of these families also showed changes in expression upon exposure to cold, suggesting they also have important roles in cold stress tolerance in the paper mulberry.

The ARF TFs play important roles in regulating diverse biological processes, including development, growth, cell division and responses to environmental stimuli [49,50]. *AtGRF5* is required for the development of appropriate leaf size and shape through the promotion and/or maintenance of cell proliferation activity in leaf primordial [51]. Cold stress inhibits shoot-ward Auxin transport and alters the intracellular Auxin gradient [52]. However, knowledge about its role under cold stress is limited. A total of 11 up-regulated and 4 down-regulated ARFs might function as the positive and negative regulator by sensing the Auxin signal in the paper mulberry under cold stress.

BBX (another abbreviation form of DBB) proteins are key factors in regulatory networks controlling growth and developmental processes that include seedling photomorphogenesis, photoperiodic regulation of flowering, shade avoidance, and responses to biotic and abiotic stresses [53]. Their functions are not entirely redundant, as judged by the fact that some DBBs were apparently implicated in light signal transduction in a negative manner, whereas another was implicated in a positive manner with regard to light-induced inhibition of elongation of hypocotyls [54]. For instance, BBX25 and BBX24 function as transcriptional co-repressors, forming inactive heterodimers with HY5 (bZIP) that down-regulate *BBX22* expression for fine-tuning of light-mediated seedling development [55]. Therefore, five *DBB*s are thought to play regulated roles, respectively in the photomorphogenesis of the paper mulberry under cold stress.

*G2-like* (GOLDEN2-LIKE) TFs are required for chloroplast development and have been reported to co-regulate and synchronize the expression of a suite of nuclear photosynthetic genes and thus act to optimize photosynthetic capacity in varying environmental and developmental conditions [56]. Although no study has reported that G2-like are related to cold stress, fifteen *G2-like* TFs were induced and eight had repressed expression exposure to cold, which suggests they may function to regulate chloroplast development in the paper mulberry under cold stress.

The LBD family is of plant-specific and implicated in plant development. Two members of the Arabidopsis LBD family, *LBD30* and *LBD18* are expressed in immature tracheary elements (TEs), and their expression is dependent on VND6 and VND7, which are NAC family TFs required for TE differentiation. *ASL20* appears to be involved in a positive feedback loop for *VND7* expression that regulates TE differentiation-related gene [57]. Four *LBD* genes downstream of ARFs, *LBD16*, *LBD17*, *LBD18* and *LBD29*, are rapidly and dramatically induced by callus-inducing medium; LBD as key regulators in the callus induction process, thereby establishing a molecular link between Auxin signaling and the plant regeneration program [58]. In addition, bHLH048 post-translated regulates the function of LOB (LBD) at lateral organ boundaries [59]. However, TCP TFs play a pivotal role in the control of morphogenesis of shoot organs by negatively regulating the expression of boundary specific genes, including *LBD*s [60]. There are 17 LBDs highly expressed in the leaf of the paper mulberry under cold stress and this might account for the advanced lateral organ development in the paper mulberry even in the cold or other hostile environment.

The WOX TFs have been identified to function in SAM (shoot apical meristem) and RAM (root apical meristem) by a dynamic feedback loop involving the CLAVATA3 (CLV3) peptide ligand and the CLV1 receptor in SAM [61]. The *dwt1* tillers have shorter internodes with fewer and un-elongated cells compared with the wild type; further study showed that the DWT1 (homologous to the Arabidopsis WOX8 and WOX9) activity in the internode elongation is directly or indirectly associated with GA (Gibberellin) signaling, suggesting a new function of WOX genes in balancing branch growth in rice [62]. Overexpression of *WOX3* induced ectopic expression of *Knotted 1‑like homeobox1* (*KNOX1*) genes in leaves and consequently produced a phenotype similar to plants ectopically expressing *KNOXI* genes [63]. However, *HOS9*, encoding the WOX6, is important for growth and development, and for a part of freezing tolerance, by affecting the activity of genes independent of the CBF pathway in Arabidopsis [64]. This is the only report that WOX TFs are involved in the cold response. In our study, three of five genes encoding WOX TFs were induced by cold exposure. Up-expression of these genes implied that they might have important roles in cambial meristems during leaf growth and development of the paper mulberry under cold stress.

Thus, TFs of the ARF, DBB, G2-like, LBD, WOX, YABBY and other families, interacted with Auxin, GA and other various hormones, were thought to be essential for the regulation of photo-morphogenesis, growth and development of the paper mulberry under cold stress.

### Clustering of the differentially expressed TFs and the identification of key TFs

**Figure 5:**
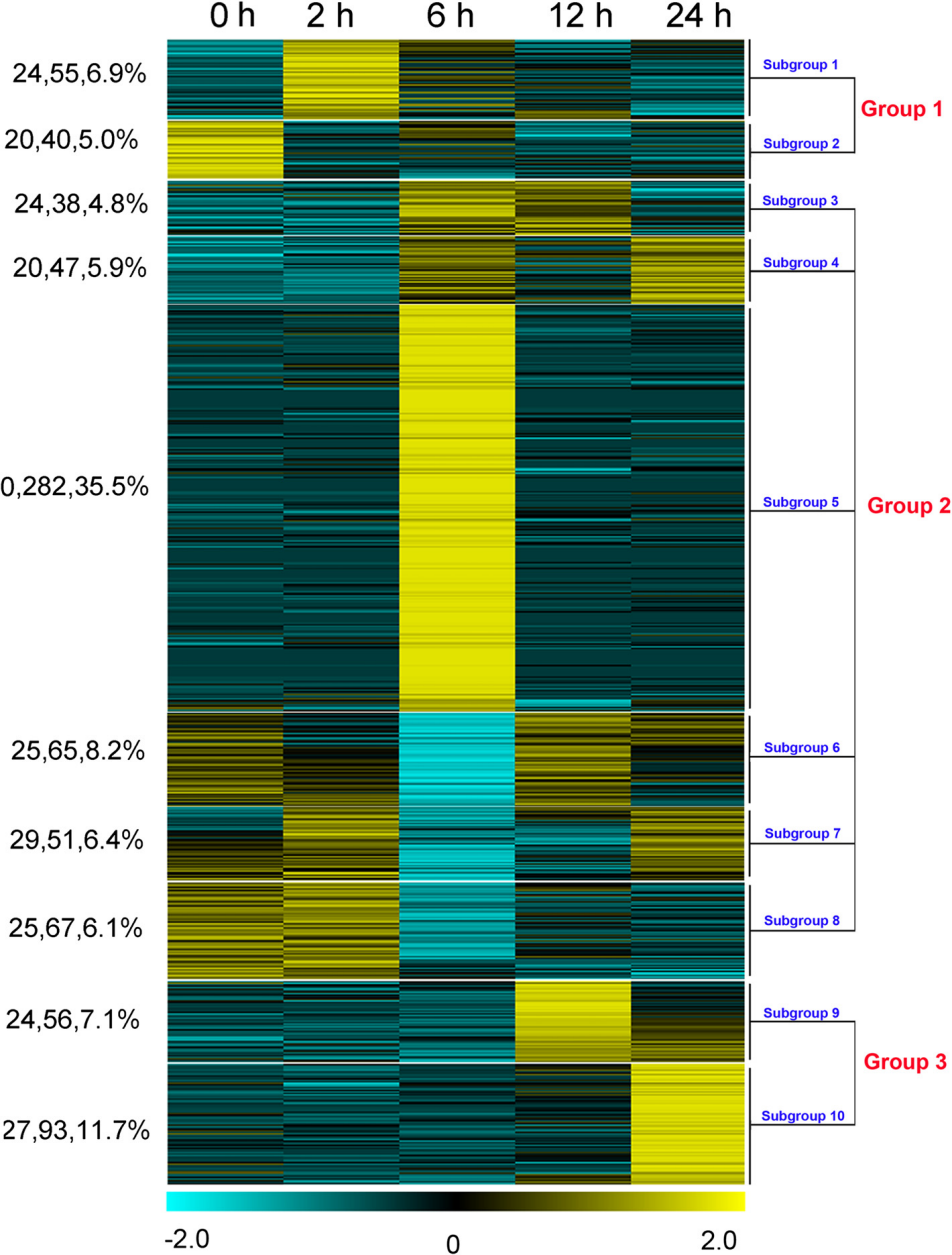
Heat map of differentially expressed TFs under cold stress. To be considered differentially expressed, the transcript must have RPKM ≥ 2 in at least one tissue, 2-fold or greater change, and P ≤ 0.05. According to their expression pattern under cold stress, the differentially expressed TFs were categorized in to 3 groups and 10 subgroups. The number at the left of the heat map (such as 24, 55, 6.9%) represented the amount of the families, differentially expressed TFs and the percentage of the total differential expressed TFs in each group, respectively. Yellow indicates high expression, black indicates intermediate expression, and blue indicates low expression.

Discerning the expression pattern and the regulatory cascades of the TFs will be benefit for the identification of key TFs that mediating the activation of downstream genes during cold stress from 0 to 24 hours in the paper mulberry. So, according to the expression profiles, the 794 differentially expressed TFs were clustered into three groups, namely early, intermediate and late responsive groups (Figure 5). Besides, the key TFs was screened from these three groups following the threshold: fold change was more than ten or the difference value of RPKM was more than 300.

**Figure 6:**
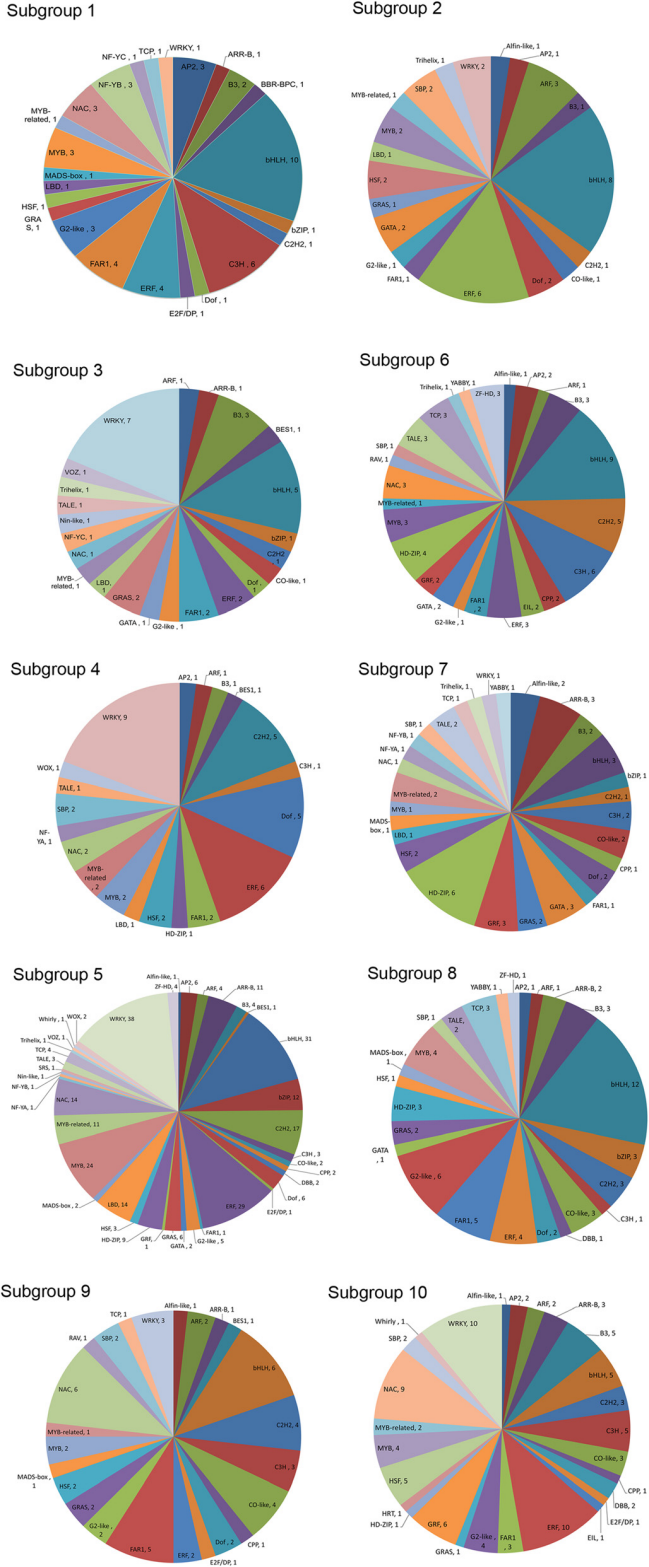
The family composition of each subgroup in the heat map. The number represented the amount of the differentially expressed TFs in each family.

TFs in group 1 was early responsive to cold stress at 2 hour, including 55 up-regulated (Subgroup 1) and 40 down-regulated (Subgroup 2). Subgroup 1 is characterized by a rapid induction of expression at 2 hours of cold treatment, followed by a slight decrease in expression for the duration of the experiment. This group contained 55 TF accounting for 6.9% of the total. A total of 40 TFs in subgroup 2 clearly underwent transcriptional repression as the cold stress began (Figure 6). Among of these 95 TFs, one up-regulated *bHLH* (T4-18787), and two down-regulated *ERF*s (T6-23630 and T7-28635) were considered to playing key role in the early activation of downstream genes. In addition, according to the results of qPCR, 3 *CAMTA*s were highly induced to express under cold stress at 15 min or 30 min (Additional file 5), though they were not detected in the transcriptomic data. Even though, this was consistent with the fact that Ca^2+^ is the second message of cold stress and CAMTA is one of the pathways that senses cold stress downstream of Ca^2+^ signal transduction. Because the transduction of Ca^2+^ signal is rapid and the balance of intracellular calcium concentration is critical, the induced expression of *CAMTA* is always fast and transient [65]. Thus, three *CAMTA*s were also considered to be the primary key TFs that sensed and transduced the cold stress signal.

The intermediate group (group 2) contained 550 differentially expressed TFs that obviously began in response to cold stress since 6 hours. According to the expression profile, group 2 has been divided into 6 subgroup, of which group 3, 4 and 5 was up-regulated while group 6, 7 and 8 was down-regulated (Figure 6). A total of 38 TFs were categorized as subgroup 3. These genes remained relatively stable through 2 hours after induction of cold stress, but increased suddenly at 6 hours and remained at heightened expression until 12 hours of cold stress. Then the expression gradually decreased to the level of untreated leaves after 24 hours treatment. Subgroup 4 contained a total of 47 TFs that were induced at 6 hours treatment of cold exposure, but the expression of these genes was decreased again by 12 hours of treatment, though their expression levels were relatively high again at 24 hours of treatment. The members of subgroup 5 were up-regulated rapidly at 6 hours treatment and then sharply declined. It contained 282 TF and was the largest subgroup, representing 35.5% of the total.

A total of 65 TFs are contained in subgroup 6. These genes have a gradual decrease in expression until 6 hours of exposure to cold, at which point they reached their lowest level of expression. At 12 hours of treatment, they reached their highest expression levels and then gradually returned to the pretreatment or slightly lower than pre-treatment levels by 24 hours. In subgroup 7, which contains 51 TFs, expression levels began to rise or slightly fluctuate from 0 to 2 hours cold stress treatment. They were then reduced to the lowest levels at 6 hours of treatment. After that, expression gradually increased and returned to relatively high levels by 24 hours of cold exposure. Unlike the TFs in subgroup 6 and 7, those showing subgroup 8 expression profiles remained relatively stable after suppressed expression at 6 hours of cold stress. This subgroup includes 67 TF belonging to 25 families.

In group 2, total 83 TFs were considered to be the key TFs mediating downstream genes in the intermediate stage of cold stress (Additional file 6). Among of them, there were 52 TFs were induced expression, including 5 bHLH, 14 ERFs, one HSF, 4 MYBs, 3 NACs and 11 WRKYs, which mainly functioned in abiotic stress response [12,22,38,43]. Meanwhile, total 31 TFs were significantly suppressed expression, such as ARR-B, B3, 5 bHLHs, 2 C2H2, 4 CO-like, 2 ERF, 3 HD-ZIP, 3 YABBYs, G2-like, GATA, GRAS and TCP. Many of these TFs have been reported play important roles in growth and development of lateral organ or apical meristem [66-68] as well as the photosynthesis [56]. These result suggested that, at the intermediate stage under cold stress, low temperature began to repress the development and growth of vegetative organ in the paper mulberry. Simultaneously, the paper mulberry initiated more stress related genes to enhance the cold tolerance.

Group 3, namely late responsive group, contained 149 TFs that were clustered into subgroup 9 and 10. These two subgroups were significantly up-regulated at the 12 and 24 hours of treatment, respectively. A total of 56 TFs were in subgroup 9 and belonged to 24 TF families accounting for 7.1% of the total. There were 93 TFs in subgroup 10, belonging to 27 families and accounting for 11.7% of the total. In late responsive group, total 18 TFs were defined as the key TFs functioning in the terminal stage because of their great changes of RPKM value just beginning from 12 or 24 hours cold stress (Additional file 6), including 3 ARR-B, C3H, 6 CO-like, 2 G2-like, 2 HSFs, 2 NACs and TCP. Most of them presented the up-regulated expression, which implied that the expression level of a large number of TFs went back to the originally expressed status and only a few of them were highly expressed to be responsible for the recovery growth and continuous cold stress.

**Figure 7:**
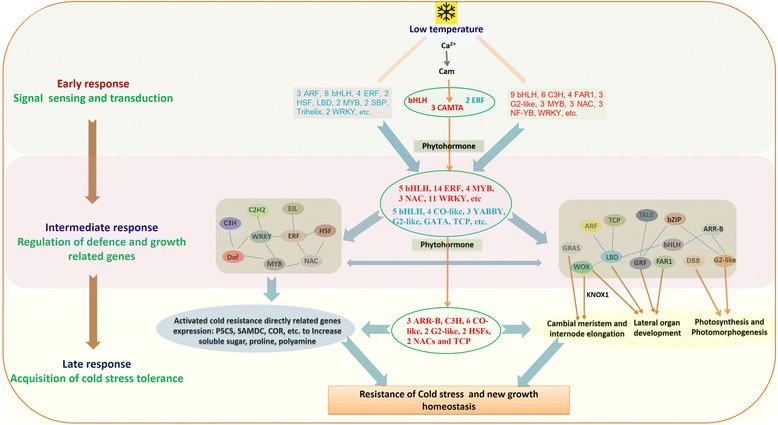
The supposed regulated network under the control of TFs of the paper mulberry exposed to cold stress. After signal reception, stress-activated Ca^2+^ signaling and other signaling modulate the expression of stress-responsive transcription factors. The thick arrows show different biological processes in the stress response under the control of TFs. The key TFs was included in the ellipse drawing by green line. Blue front TFs represented the repressed expressed TFs and the red front TFs represented the induced expression TFs. The blue line between TFs showed in the intermediate response group presented the existed the interaction among these TFs.

Based on the above differential expression analysis, the proposed cold response regulatory cascades under the regulation of TFs in the paper mulberry was illustrated in Figure 7. After signal reception, stress-activated Ca^2+^ signaling and other secondary signal modulate the expression of early cold stress responsive TFs. One bHLH, two ERFs and three CAMTAs played the key roles in the primary signal transduction. After that, at the intermediate stage of cold stress, there were mainly two biological processes that were regulated by TFs, namely cold stress resistance and the growth homeostasis. On the one hand, much cold stress related genes could be repressed or induced by TFs, such as WRKY, HSF, ERF, NAC, MYB, etc., which played the central roles in endowing paper mulberry with the cold tolerance by regulating the content of osmoprotectant, including soluble sugar, proline and so on. On the other hand, the major growth physical process, e.g. photosynthesis, chloroplast malfunction, cell division in cambial meristem and lateral organ development, were mainly regulated by YABBY, WOX, LBD, DBB, GRF, etc., which made the growth state of paper mulberry reach to a new homeostasis. Of cause, the crosstalk and the intricate regulation network could exist in these processes. At the late stage of cold stress, the expression of most TFs restored to the level before cold stress. A few of TFs, mainly concentrated in the ARR-B, CO-like, G2-like and TCP families, were drastically induced expression to respond to the new growth homeostasis under low temperature stress.

## Conclusions

Our study is the comprehensive transcriptomic-wide identification of cold stress-responsive TFs in the paper mulberry. Total of 794 TFs belonging to 47 families were responded to cold stress and they were clustered into three groups, namely early, intermediate and late responsive groups. The key TFs were also screened as playing the important roles in three stages. In addition, based on the analysis, the AP2/ERF, bHLH, MYB, NAC and WRKY families play central and significant roles during cold stress response in the paper mulberry just as in other species, as many members of these families are up-regulated during exposure to cold. The finding indicates a substantial conservation of TF families that regulate cold stress response across different species of plant. In addition, we identified many other TF families previously associated with growth and development, including ARF, DBB, G2-like, GRF, GRAS, LBD, WOX and YAABY, which exhibit obviously altered expression under cold stress in the paper mulberry. Although it seemed that their important roles in the cold stress response have been neglected, these proteins might contribute to the regulation of cold response in this species. This study provides us the profile of the TFs involved in the cold stress adaptation of the paper mulberry and also highlights that many plant growth and development related TFs are affected by cold stress, suggesting the potential of using these TFs to improve the plant growth under cold conditions instead of simply enhancing the cold stress tolerance.

## Methods

### Plant material and RNA extraction

Plantlets were cultured on MS culture media in an artificial climatic chamber kept at 26°C with a 14/10 h photoperiod (day/night). For low temperature treatment, plantlets were grown as above, and transferred to 4°C. Leaves were sampled at different time points (0, 2, 6, 12 and 24 h). In this study, a mixed sampling strategy was adopted to eliminate differences between individuals.

Total RNA was isolated with TRIzol® Reagent (Life Technologies, Shanghai, China) from each sample according to the manufacturer’s instructions. It was treated with RNase-free DNase I (Takara, Dalian, China) to remove the residual DNA. RNA quality and purity were assessed with OD260/230 ratio and RNA integrity number (RIN) by using the NanoDrop 2000 (Thermo Fisher, Waltham, USA) and the Agilent 2100 Bioanalyzer (Agilent Technologies, Santa Clara, USA), respectively.

### cDNA library preparation, sequencing (RNA-seq) and transcriptome assembly

The libraries were prepared according to former study [69]. After clustering on a flow cell using the cBOT, the cDNA libraries were loaded on the Illumina Genome Analyzer ΠX platform and sequenced. Raw sequence data were generated by Illumina pipeline and were available in NCBI’s Short Read Archive (SRA) database (http://www.ncbi.nlm.nih.gov/Traces/sra/sra.cgi) under accession number SRP029966. All of the Illumina reads generated from cDNA libraries were pooled together and *de novo* assembled with the Trinity program to form the global transcriptome of the paper mulberry.

### Annotation, classification and expression analysis of TFs

For functional annotation, unigenes were firstly aligned by Blastx to protein databases NCBI nr, Swiss-Prot, TrEMBL, KEGG and COG. A significance cut off of E ≤ 1e-5 was used to ensure that only the proteins with the highest sequence similarity to the given unigenes were retrieved, along with their functional annotations. After getting annotation result for every unigene, all of the TFs of the paper mulberry were identified and classified into different families based on their DNA-binding domains and other conserved motifs [70,71]. In addition, the TF families’ abbreviations were referenced to PlantTFDB 3.0 (http://planttfdb.cbi.pku.edu.cn/index.php) and PlnTFDB (3.0) (http://plntfdb.bio.uni-potsdam.de/v3.0/). All of these TF sequences information were provided in Additional file 1.

For gene expression analysis, the expression level of each TF in each sample was calculated by quantifying the number of Illumina reads that mapped to transcriptome of the paper mulberry with default parameters. The raw gene expression counts were normalized using the RPKM method (Reads per kb per million reads).

### Identification of differentially expressed TFs in response to cold stress

For screening of differentially expressed TFs, p value corresponds to differentially expressed genes (DEGs) was obtained via a general Chi squared test that was performed by using IDEG6 (http://telethon.bio.unipd.it/bioinfo/IDEG6/). The threshold of p value in multiple tests was checked through manipulating the false discovery rate (FDR) value. The TFs with a ratio of RPKM between samples of more than 2 (Fold change ≥ 2 or ≤0.5) and an FDR ≤0.01 were considered to have significant changes in expression in response to cold stress. The Multiexperiment Viewer (v4.9) was used to make the heat map and expression classification.

### Validation by qPCR

The qPCR was adopted to validate the DEGs identified in analysis of the RNA-seq data. Ten TFs were chosen for verification. RNA used for validation was the same as that isolated for RNA-seq. In addition, two samples (samples were kept under cold stress for 15 min and 30 min) were added to detect the early expression profile of CAMTA TFs. First-strand cDNA for each sample was made from 1 μg of total RNA using SuperScript II reverse transcriptase (Takara, Dalian, China) following the manufacturer’s recommendations and diluted 3 times before use in PCR. Gene-specific primers based on the selected considerate unigenes were subsequently designed using the Primer Premier 5 program and are listed in Additional file 7. QPCR reaction conditions and volume was performed as described by our former study [69]. Relative transcript levels for each sample were obtained using the comparative cycle threshold method using the cycle threshold value of the *actin* gene for each sample as a control.

### Availability of supporting data

The data set supporting the results of this article is available in the NCBI’s Short Read Archive (SRA) database (http://www.ncbi.nlm.nih.gov/Traces/sra/sra.cgi) under accession number SRP029966.

## Additional files

Additional files 1:
**The sequence information and expression values of the TFs expressed in these five samples.**
Additional files 2:
**The expressed and the differently expressed TFs distributed in every family in each sample.** A The expressed TFs statistic in every family in each sample B The differentially expressed TFs statistic in every family in each sample. Additional files 3:
**The differentially expressed TFs with complete ORF distributed in every family.**
Additional files 4:
**The expression values of the differentially expressed TFs in these five samples.**
Additional files 5:
**The expression profile of ten selected differentially expressed TFs validated by qPCR.** The left axis represents the results of transcriptomics analysis while the right axis represents relative expression detected by qPCR. Additional files 6:
**The key TFs identified in the paper mulberry under cold stress.**
Additional files 7:
**The selected TFs and their primer for qPCR.**

## Abbreviations

ABA: Abscisic acid
CBF: C-repeat binding factor
ESTs: Expressed sequence tags
GA: Gibberellin
JA: Jasmonic acid
ORF: Opening read frame
RAM: Root apical meristem
RPKM: Reads per kb per million reads
SA: Salicylic acid
SAM: Shoot apical meristem
SRAP: Sequence-related amplified polymorphism
TF: Transcription factors
