# Supplementary figures and images for: Transcriptional regulation of the paper mulberry under cold stress as revealed by a comprehensive analysis of transcription factors
Source: BMC Plant Biol. 2015 Apr 19;15:108. doi: 10.1186/s12870-015-0489-2 (PMC4432934; doi:10.1186/s12870-015-0489-2)

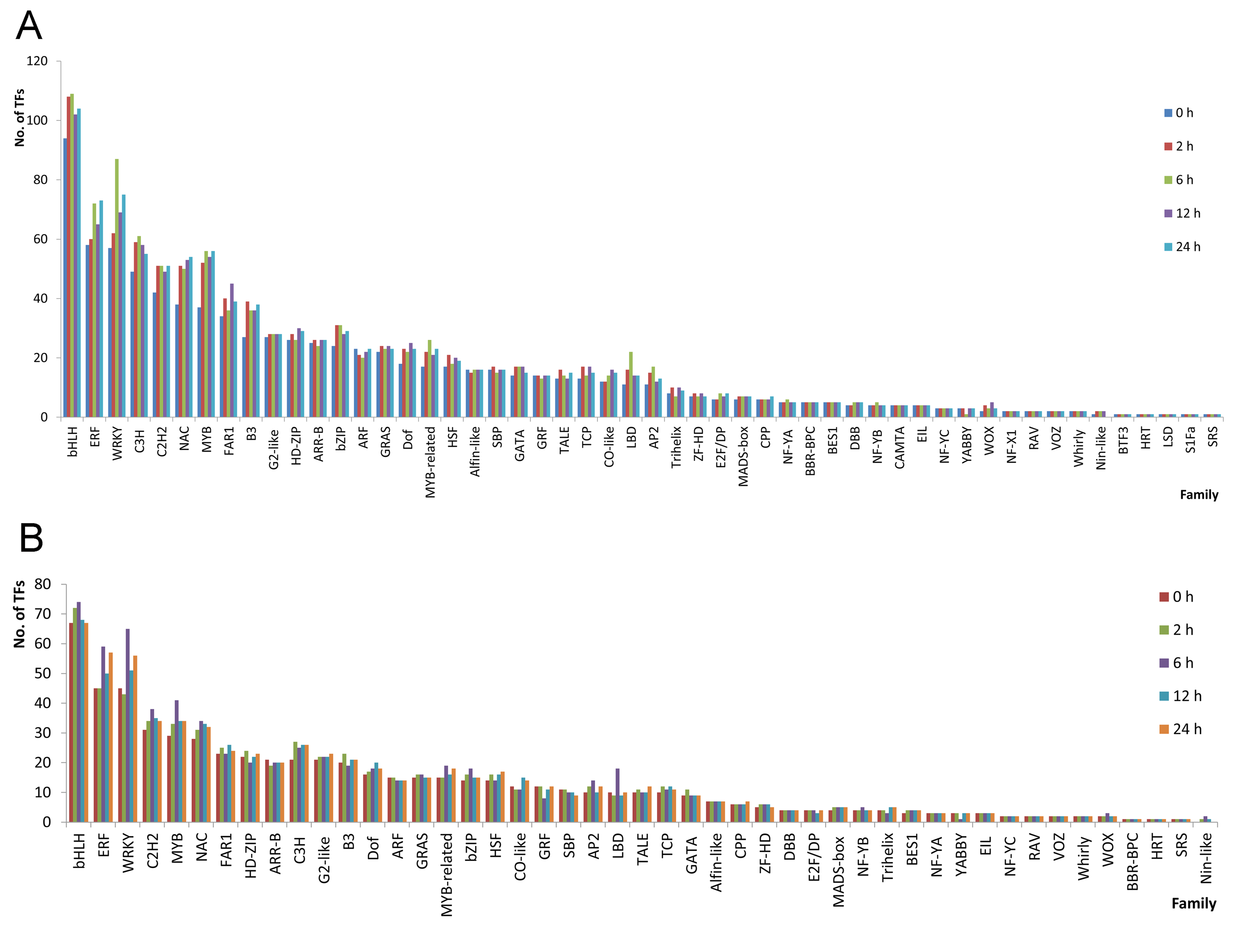

Supplement: Additional files 2: — The expressed and the differently expressed TFs distributed in every family in each sample. A The expressed TFs statistic in every family in each sample B The differentially expressed TFs statistic in every family in each sample. [file 12870_2015_489_MOESM2_ESM.tiff]

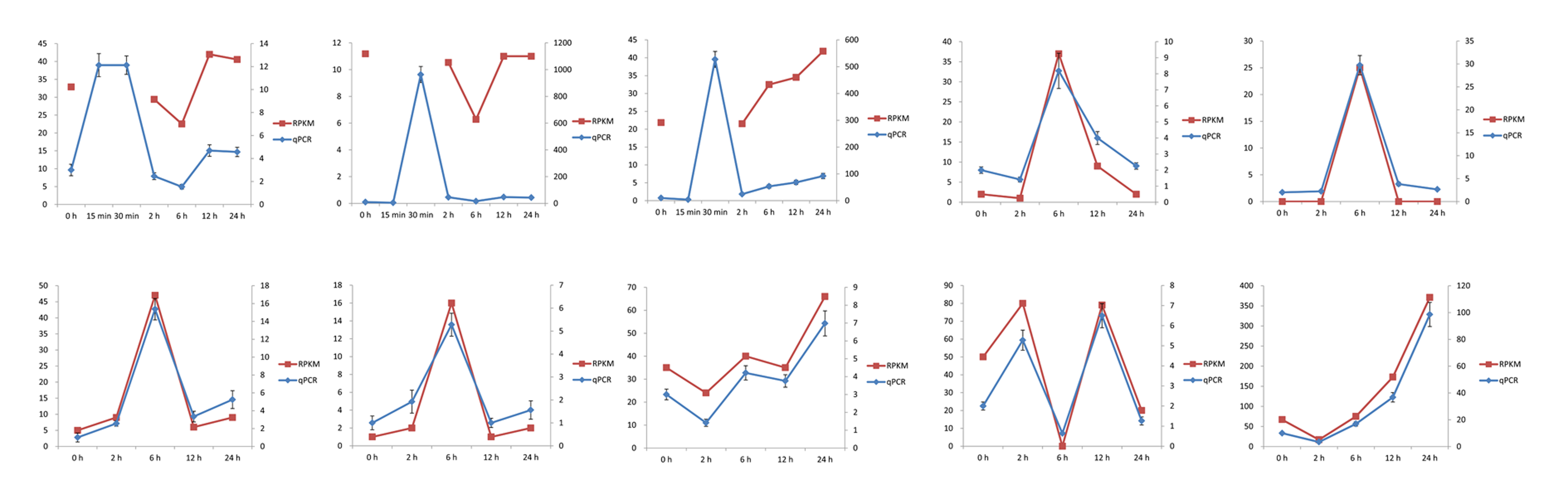

Supplement: Additional files 5: — The expression profile of ten selected differentially expressed TFs validated by qPCR. The left axis represents the results of transcriptomics analysis while the right axis represents relative expression detected by qPCR. [file 12870_2015_489_MOESM5_ESM.tiff]
