# Supplementary material for: Transcriptional regulation of the paper mulberry under cold stress as revealed by a comprehensive analysis of transcription factors
Source: BMC Plant Biol. 2015 Apr 19;15:108. doi: 10.1186/s12870-015-0489-2 (PMC4432934; doi:10.1186/s12870-015-0489-2)
Supplement: Additional files 7: — The selected TFs and their primer for qPCR. [file 12870_2015_489_MOESM7_ESM.docx]

| TF family | ID of selected TF | Name of Primer | Primer Sequence 5' to 3' |
| --- | --- | --- | --- |
| ARF | T5-27643 | Bp1-F | AGCCAAGATGTTCAGTCTCAGATAACC |
|  |  | Bp1-R | GTCATTAAGCAGCCCTTCAAGTCC |
| ARR-B | T3-14633 | Bp2-F | GCTTGATGGCGAGAAGAACTCC |
|  |  | Bp2-R | TTCAATATGAACTCCTCCGCACC |
| CAMTA | T6-28524 | Bp3-F | GAAATGCCAGATGCACTGTCG |
|  |  | Bp3-R | GCAGAGTGAGCTAACCCATCACC |
| CAMTA | T7-28477 | Bp4-F | TCAAGTTCGCAAGCAGTATAAAAAGC |
|  |  | Bp4-R | TCTCATTGGTTATTAGCAAATGCAGG |
| CAMTA | T7-32553 | Bp5-F | TAAAGGAAGGTAGGAAGCAATACGAGG |
|  |  | Bp5-R | TAAAAGTGTCATCGTCCAACAAAGC |
| EIL | T7-31855 | Bp6-F | TGCAAATGATGCAAGGTAATATCTGG |
|  |  | Bp6-R | CAGGCACCTCTCTTTCACATGC |
| GATA | T3-23795 | Bp7-F | TTTCCCTCAAGACGAGAAAGACG |
|  |  | Bp7-R | AGCAGATGCTAAGGAACAAGAGATCC |
| LBD | T5-25115 | Bp8-F | AAAATGCGGATGAGCTGTAATGG |
|  |  | Bp8-R | GTACAAAAGCGACCGAAAAATCG |
| MYB | T5-24338 | Bp9-F | TCGGAACAAACAGGAAAATCTCC |
|  |  | Bp9-R | CCTCGTTCATTAAAAAACCATGTCC |
| WRKY | T2-21870 | Bp10-F | CGAGCATCCCATGATCTAAGAGC |
|  |  | Bp10-R | TTTCCAAACCCTGCATTGAACC |

Additional file 7 The selected TFs and their primer for qPCR
